# Synthesis, characterization, and crystal structure of hexa­kis­(1-methyl-1*H*-imidazole-κ*N*^3^)zinc(II) dinitrate

**DOI:** 10.1107/S2056989024008806

**Published:** 2024-09-24

**Authors:** Nomampondo Penelope Magwa, Thompho Jason Rashamuse

**Affiliations:** a University of South Africa, Department of Chemistry, Private Bag X6, Florida, Gauteng, 1710, South Africa; b Advanced Materials Division, Mintek, 200 Malibongwe Drive, Randburg, 2125, South Africa; University of Massachusetts Dartmouth, USA

**Keywords:** *N*-methyl­imidazole, Zn complex, NMR, FTIR, crystal structure

## Abstract

The synthesis of the title compound, [Zn(C_4_H_6_N_2_)_6_](NO_3_)_2_, is described. This complex consists of a central zinc metal ion surrounded by six 1-methyl­imidazole ligands, charge balanced by two nitrate anions. The complex crystallizes in the space group *P*. In the crystal, the nitrate ions are situated within the cavities created by the [Zn(*N*-Melm)_6_]^2+^ cations, serving as counter-ions. The three oxygen atoms of the nitrate ion engage in weak C—H⋯O inter­actions. In addition to single-crystal X-ray diffraction analysis, the complex was characterized using elemental analysis, ^1^H NMR, ^13^C NMR, and FTIR spectroscopy.

## Abstract 2

The title complex, which consists of a central zinc metal ion surrounded by six 1-methyl­imidazole ligands, charge balanced by two nitrate anions and which crystallizes in the space group *P*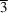 has been synthesized and its structure determined.

## 1. Chemical context

Extensive research has been conducted on zinc complexes containing imidazole and its derivatives due to their significance in chemistry and their diverse applications (Victor *et al.*, 2014 ▸; Porchia *et al.*, 2020 ▸). These complexes play crucial roles as anti­cancer agents (Porchia *et al.*, 2020 ▸; Babijczuk *et al.*, 2023 ▸), anti­bacterial agents (Guo *et al.*, 2022 ▸), fluorescent sensors (Anjali *et al.*, 2022 ▸), in anti-counterfeiting and latent fingerprint detection (Kempegowda *et al.*, 2021 ▸), and in materials chemistry (Rashamuse *et al.*, 2023 ▸; Bezvikonnyi *et al.*, 2022 ▸; Yu *et al.*, 2021 ▸). Notably, zinc is the second most prevalent trace metal in the human body and is essential in a variety of biological systems (Haase & Rink, 2014 ▸; Kolenko *et al.*, 2013 ▸). Consequently, it is unsurprising that Zn^II^ ions demonstrate the ability to inhibit certain bacterial species (McDevitt *et al.*, 2011 ▸; Velasco *et al.*, 2018 ▸). The use of Zn^II^ as the metal center in coordination chemistry is motivated by its ability to form strong complexes with ligands and the low cost of Zn precursors (Häggman *et al.*, 2020 ▸; Rashamuse *et al.*, 2023 ▸). In recent years, great efforts have been made to develop new organic zinc complexes with various architectures and applications (Abendrot, *et al.*, 2020 ▸; Brahma & Baruah, 2020 ▸; Chen *et al.*, 2021 ▸; Kseniya *et al.*, 2022 ▸; Loke *et al.*, 2020 ▸).

On the other hand, N-substituted imidazoles, or 1-substituted imidazoles, have emerged as highly attractive compounds due to a broad spectrum of applications (Chen *et al.*, 2020 ▸; Gu *et al.*, 2014 ▸; Kanzaki *et al.*, 2012 ▸; Kseniya *et al.*, 2022 ▸; Liu *et al.*, 2014 ▸; Bogdanov & Svinyarov, 2017 ▸; Park *et al.*, 2020 ▸; Wang *et al.*, 2013 ▸). This ligand set features a conjugated di­aza five-membered heterocyclic ring structure. One nitro­gen atom has an N-methyl substituent, and its lone pair is delocal­ized in the aromatic ring, while the other nitro­gen is *sp*^2^ hybridized and capable of coordinating Lewis acids, including metal ions. Numerous studies have been published on transition metal ion complexes involving imidazole and its derivatives (Erer *et al.*, 2011 ▸; He *et al.*, 2021 ▸; Kühl *et al.*, 2011 ▸; Jawad & Al-Adilee 2022 ▸; Konarev *et al.*, 2018 ▸; Neumüller & Dehnicke, 2010 ▸; Reedijk *et al.*, 2012 ▸; Zhang *et al.*, 2020 ▸). The lack of N-methyl group tautomerization enhances the appeal of N-substituted imidazole for the synthesis of novel mol­ecules. The coordination of imidazole derivatives with metal centers has had a positive impact on the development of novel metal complexes with applications in the field of material science (Anjali *et al.*, 2022 ▸; Bezvikonnyi *et al.*, 2022 ▸; Kempegowda *et al.*, 2021 ▸; Rashamuse *et al.*, 2023 ▸; Yu *et al.*, 2021 ▸). In addition, the use of imidazole derivatives alongside zinc metal ions is an inter­esting technique to expand the complex repertoire in coordination chemistry. Therefore, our goal was to utilize the *N*-methyl­imidazole core in conjunction with a zinc metal ion in the presence of ammonia to generate unique complexes with different topologies.
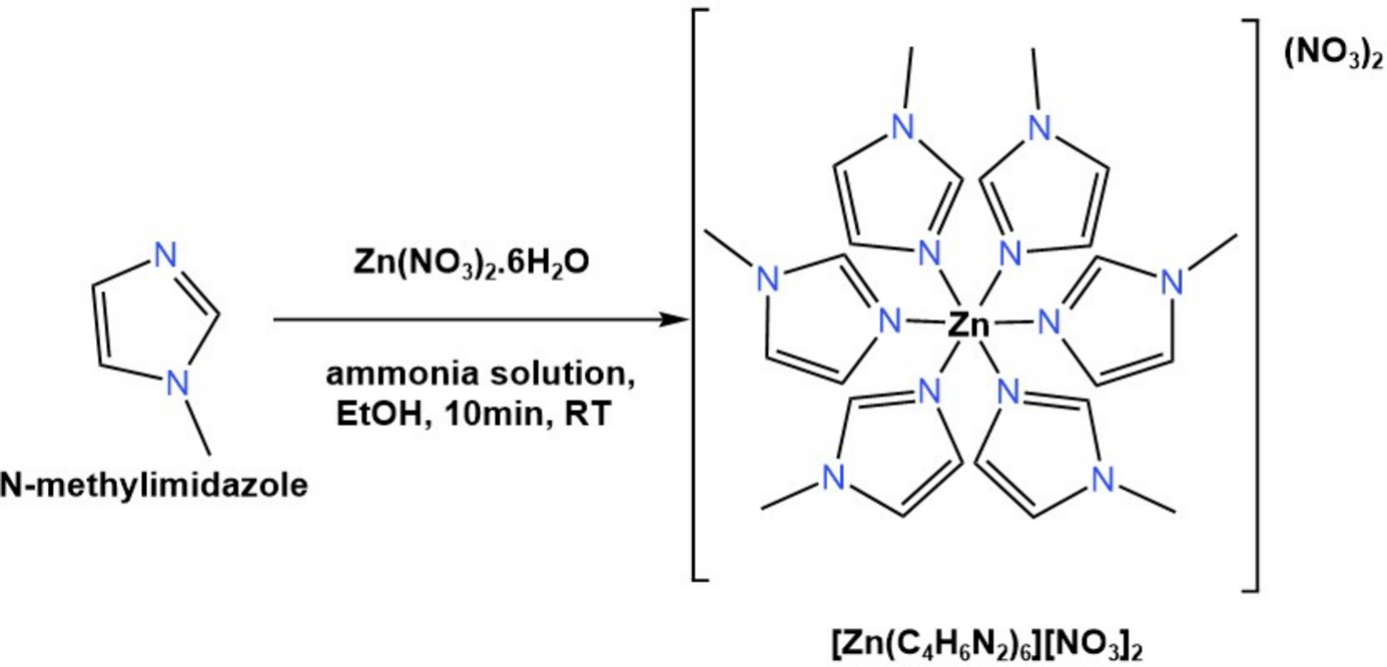


The utilization of 1-methyl­imidazole as a starting ligand for the synthesis of zinc complexes has been previously explored (Rashidi *et al.*, 2021 ▸; Appleton & Sarkar, 1977 ▸; Chen *et al.*, 1996 ▸; Steichen *et al.*, 2014 ▸). In this article, we report the synthesis of a new compound hexa­kis­(1-methyl-1*H*-imidazole-κ*N*^3^)zinc(II) dinitrate, [Zn(C_4_H_6_N_2_)_6_](NO_3_)_2_, which is synthesized in the manner depicted in the scheme. The structure of the complex was confirmed *via* proton NMR, FTIR, and single-crystal X-ray diffraction.

## 2. Structural commentary

**Figure 1:**
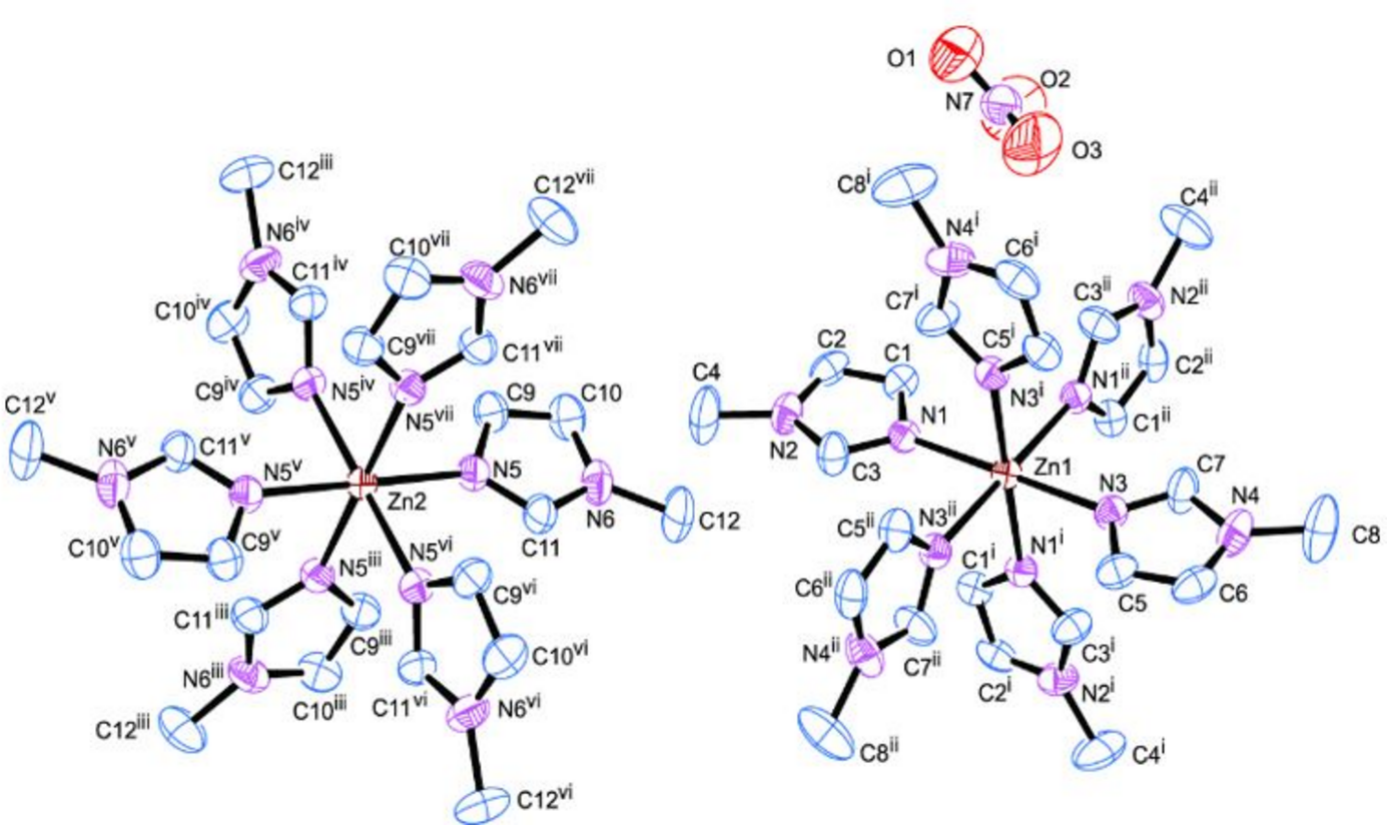
Displacement ellipsoid plot of [Zn(C_4_H_6_N_2_)_6_][NO_3_]_2_ showing the atom-numbering scheme. Displacement ellipsoids are drawn at the 50% probability level. [Symmetry codes: (i) *y* − *x*, 1 − *x*, *z*; (ii) 1 − *y*, 1 + *x* − *y*, *z*; (iii) −*y*, *x* − *y*, *z*; (iv) *y* − *x*, −*x*, *z*; (v) −*x*, −*y*, −*z*; (vi) −*y*, *x* + *x*, −*z*; (vii) *y*, −*x* + *y*, −*z*.]

The title compound (Fig. 1 ▸) crystallizes in the *P*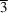 space group with half of a formula unit in the asymmetric unit. There are two crystallographically distinct zinc atoms. One has 1/3 occupancy and is bound to two crystallographically unique 1-methyl­imidazole ligands and the other has 1/6 occupancy and is bound to one crystallographically unique 1-methyl­imidazole ligand. One full occupancy nitrate anion is also present in the asymmetric unit.

The full zinc complex ions {[Zn(*N*-Melm)_6_]^2+^, where *N*-Melm denotes *N*-methyl­imidazole} exhibit coordination by six *N*-Melm ligands. The ions are in a distorted octa­hedral coord­ination environment, demonstrated by N—Zn—N angles close to 90° or 180° depending on their *cis* or *trans* relationship. The Zn—N lengths are 2.182 (2) Å for N1—Zn1, 2.177 (2) Å for N3—Zn1, and 2.179 (2) Å for N5—Zn2. The complex mol­ecule also displays fifteen unique C—N bond lengths ranging from 1.308 (3) to 1.471 (4) Å. The nitrate counter-ion demonstrates O—N—O bond angles of 125.4 (4) ° for O2—N7—O3, 118.0 (4)° for O2—N7—O1, and 116.5 (4) ° for O3—N7—O1 and N—O bond lengths of 1.202 (5) Å for N7—O2, 1.209 (4) Å for N7—O3 and 1.234 (5) Å for N7—O1.

## 3. Supra­molecular features

**Figure 2:**
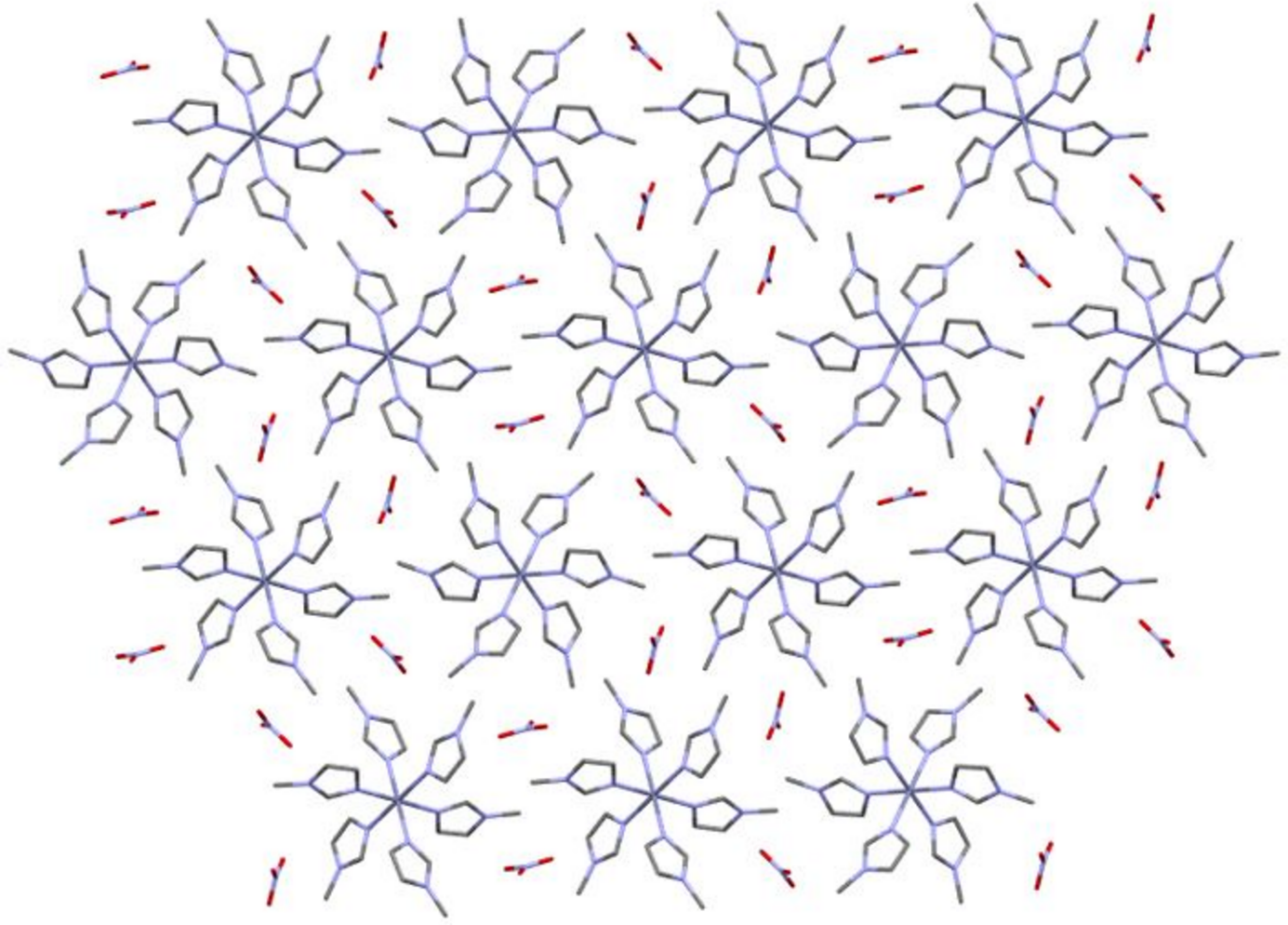
Packing diagram of the [Zn(C_4_H_6_N_2_)_6_][NO_3_]_2_ complex showing the nitrate cation lying in the void between the cationic complexes.

**Figure 3:**
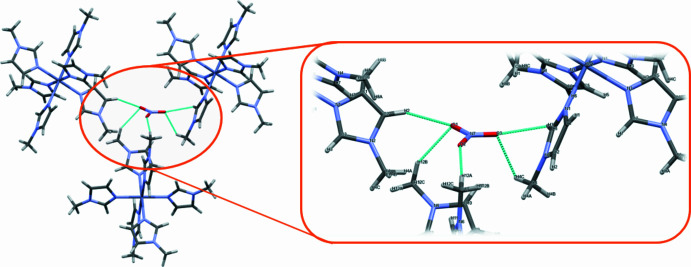
Diagram showing inter­action between the nitrate ions and *N*-methyl­imidazole ligands of the title compound with blue dashed lines representing the C—H⋯O close contacts.

The packing of the title compound is shown in Fig. 2 ▸ while Fig. 3 ▸ shows the inter­molecular inter­actions in the [Zn(C_4_H_6_N_2_)_6_][NO_3_]_2_ complex. In the crystal, the nitrate ions are situated within the cavities created by the [Zn(*N*-Melm)_6_]^2+^ cations, serving as counter-ions. The three oxygen atoms of the nitrate ion engage in weak C—H⋯O inter­actions (Table 1 ▸) with two hydrogen atoms from the imidazole rings and one hydrogen atom from the methyl groups.

## 4. Database survey

A search of the Cambridge Structural Database (CSD, Version 5.45, update of March 2024; Groom *et al.*, 2016 ▸) for [Zn(C_4_H_6_N_2_)_6_] compounds with nitrate cations resulted in no hits. However, when the search was expanded to include other cationic salts, six relevant ones were found, including a discrete Zn complex with a sixfold coordination. These entries include [Zn(C_4_H_6_N_2_)_6_](I)_2_ (CCDC reference: 2347502; Rashidi *et al.*, 2021 ▸), [Zn(MeIm)_6_](Tf_2_N)_2_, [Zn(EtIm)_6_](Tf_2_N)_2_, [Zn(MeIm)(EtIm)_5_](Tf_2_N)_2_, [Zn(MeIm)_2_(EtIm)_4_](Tf_2_N)_2_, and [Zn(MeIm)_4.5_(EtIm)_1.5_](Tf_2_N)_2_ (CCDC references: 978387, 978388, 978389, 978390, and 978391; Steichen *et al.*, 2014 ▸) where Melm is 1-methyl-1*H*-imidazole), EtIm is 1-ethyl-1*H*-imidazole, and (Tf_2_N)_2_ is bis­(tri­fluoro­methyl­sulfon­yl)imide. The cationic zinc complex paired with iodine anions, [Zn(C_4_H_6_N_2_)_6_](I)_2_, is particularly inter­esting as it exhibits a very similar structure, similar packing, similar cell parameters, and the same space group as the title compound. Although the zinc ions in the other five complexes had a similar sixfold coordination, the anion involved in these complexes was bis­(tri­fluoro­methyl­sulfon­yl)imide, and their crystals exhibited different structures and space groups.

## 5. Synthesis and crystallization

In a typical synthesis, 0.9 g of zinc nitrate hexa­hydrate, Zn(NO_3_)_2_·6H_2_O (3.0 mmol), was dissolved in 10 mL of ethanol. A second solution consisting of 0.52 g of *N-*methyl-1*H*-imidazole (6.0 mmol) in 30 mL of ethanol and 2.8 ml of ammonia solution (2.48 mmol) was prepared in parallel. The Zn^II^ solution was poured rapidly into the second solution. The resultant mixture was stirred at room temperature for 10 minutes to complete crystallization. The crystals were collected by centrifugation, filtered, washed three times with ethanol, and dried overnight at room temperature to afford [Zn(C_4_H_6_N_2_)_6_][NO_3_]_2_ as light-blue crystals in 67% yield. Analysis calculated for C_24_H_36_N_14_O_6_Zn: C, 42.27%; H 5.32%; N, 28.75%; Found: C, 42.71%; H5.39%; N, 28.56%; ^1^H NMR δ/ppm (400 MHz, CDCl_3_): 3.77 (*s*, 3H), 7.06 (*s*, 2H), 8.13 (*s*, 1H); ^13^C NMR δ/ppm (101 MHz, CDCl_3_): 32.23, 127.99, 140.04; FTIR ν_max_/cm^−1^: 3121, 1643, 1528, 1516, 1327, 1288, 1231, 1088, 1026, 934, 826,768, 660, 621.

**Figure 4:**
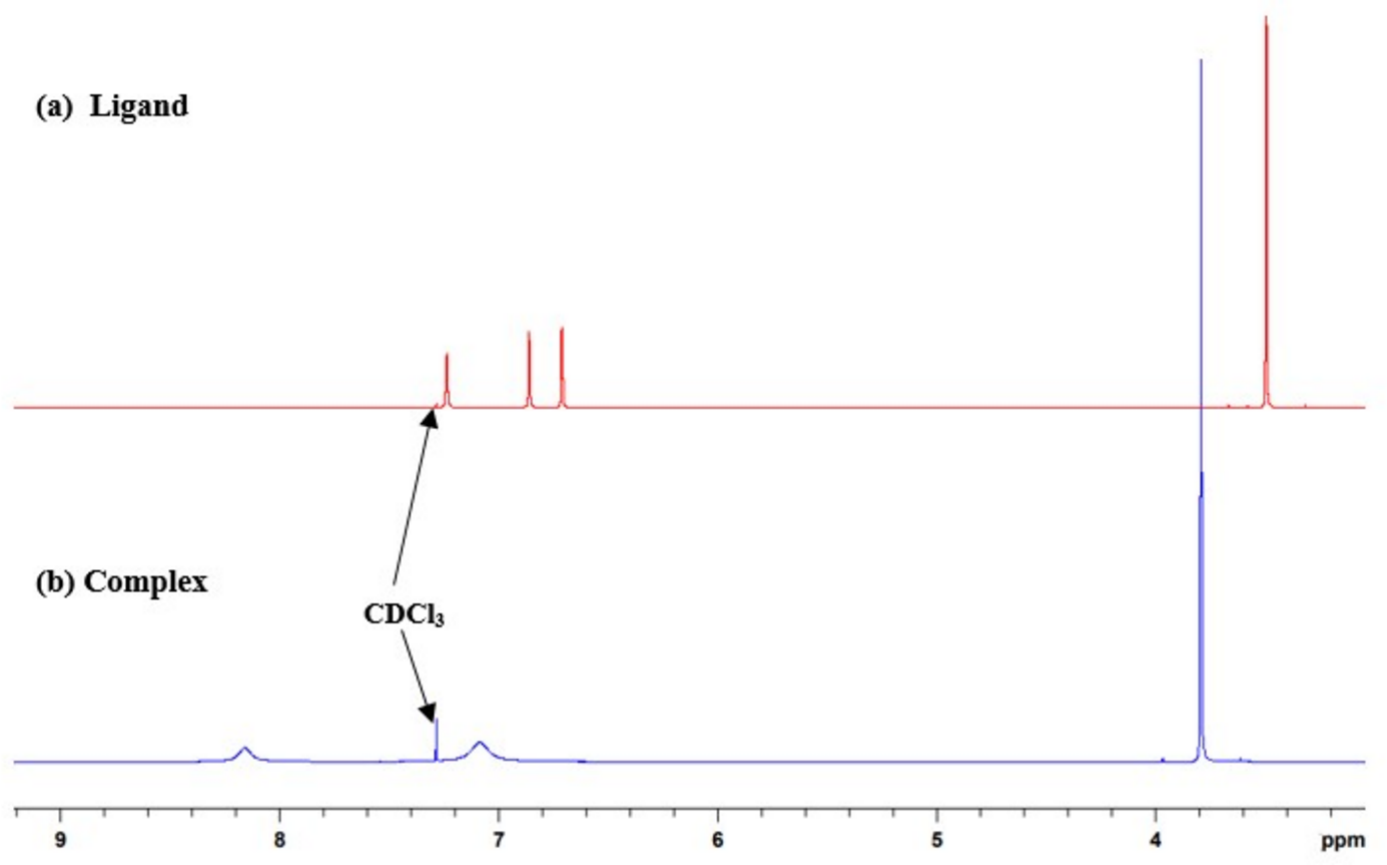
The superimposed ^1^H NMR spectra of (*a*) the free *N*-methyl­imidazole ligand (red) and (*b*) its corresponding [Zn(C_4_H_6_N_2_)_6_][NO_3_]_2_ complex (blue) in deuterated chloro­form.

The overlaid ^1^H NMR spectra of the ligand and zinc complex are shown in Fig. 4 ▸. In the proton NMR spectrum of the free *N*-methyl-1*H*-imidazole ligand, there are four sharp signals with the methyl group appearing at 3.47 ppm and the three protons of the imidazole motif appearing at 6.69. 6.84 and 7.21 ppm. However, upon complexation with the zinc ion, the methyl proton shifted to 3.77 ppm, while the imidazole signals are broadened with two protons merged at 7.06 ppm, and the third proton exhibiting a downfield shift to 8.13 ppm. Similar behavior can also be observed in the ^13^C NMR spectra, demonstrating complexation.

**Figure 5:**
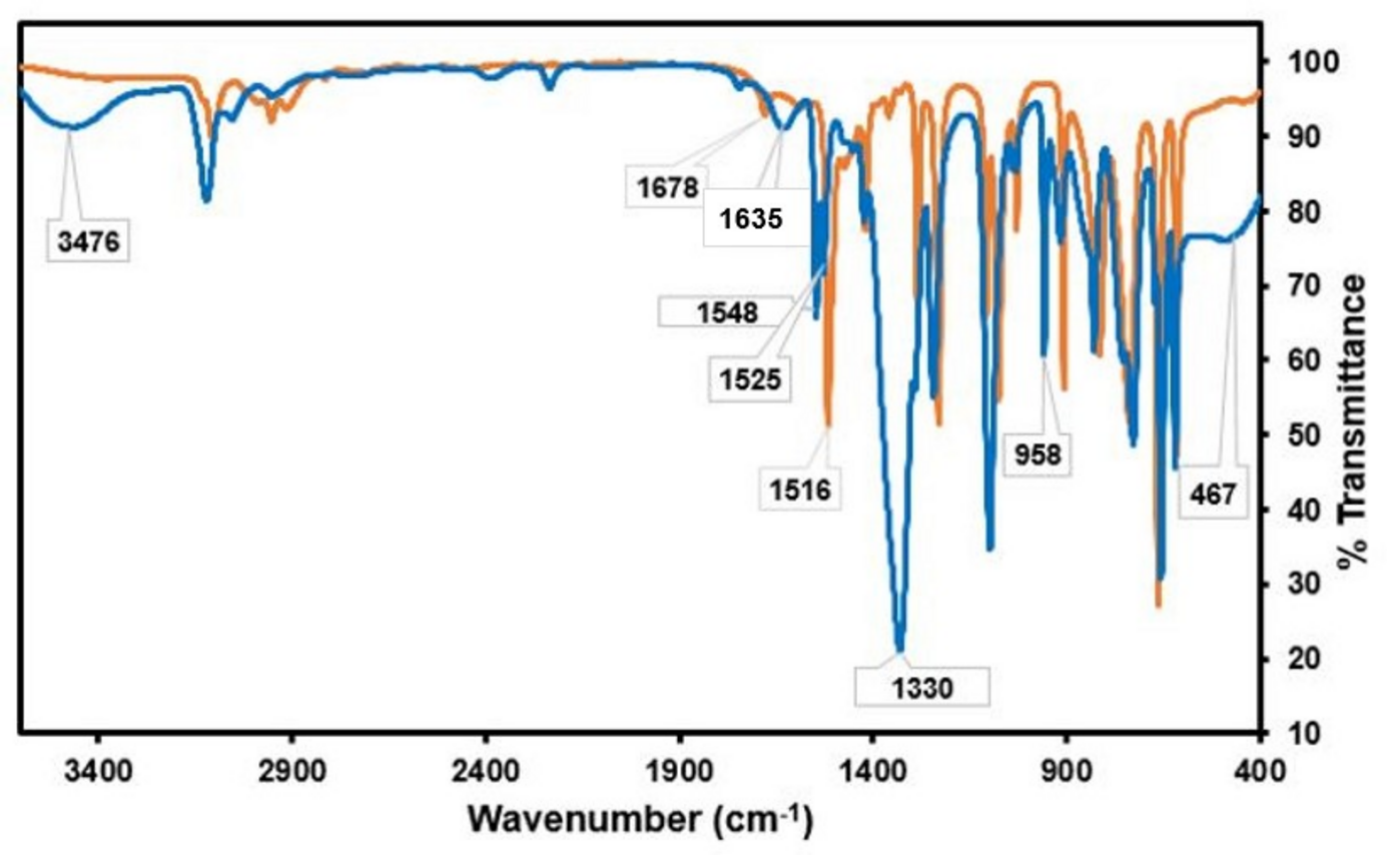
The superimposed FTIR spectra of the free ligand *N*-methyl­imidazole (orange) and its [Zn(C_4_H_6_N_2_)_6_][NO_3_]_2_ complex (blue) in the frequency range 3600–400 cm^−1^.

The comparative FTIR spectra of the free ligand and the zinc complex are presented in Fig. 5 ▸. In the spectrum of the zinc complex, a vibrational peak at 3476 cm^−1^ is observed, which is attributed to the O—H stretching of water mol­ecules as the complex is hygroscopic. The characteristic vibrational peak associated with C=C stretching appears at 1678 cm^−1^ in the free ligand, but in the complex spectrum, it is shifted to the vibrational frequency of 1630 cm^−1^. Furthermore, an intense band at around 1516 cm^−1^ is evident, which originates from the C=N stretching mode of the imidazole moiety of the free ligand. However, upon zinc coordination, two different vibrational frequencies are observed at 1545 and 1527 cm^−1^, corresponding to the C=N stretching mode. Notably, both asymmetric and symmetric NO_3_ stretching vibrations at 1330 and 958 cm^−1^ are clearly visible as intense vibrational peaks in the zinc complex spectrum, with both features absent in the free ligand spectrum. Furthermore, a stretching band of Zn—N is observed at a vibrational frequency of 467 cm^−1^, providing further evidence of the coordination of zinc ions with the nitro­gen atom of the *N*-methyl­imidazole group.

## 6. Refinement

Crystal data, data collection and structure refinement details are summarized in Table 2 ▸. All C bound hydrogen atoms were placed at idealized positions and refined as riding atoms with isotropic parameters 1.2 or 1.5 times those of their parent atoms. The crystal studied was refined as a two-component twin. H atoms were positioned in idealized locations and refined using a riding model, with isotropic displacement parameters set to 1.2 or 1.5 times those of their respective parent atoms. A twin law was also applied.

## Supplementary Material

Crystal structure: contains datablock(s) I. DOI: 10.1107/S2056989024008806/yy2011sup1.cif

Structure factors: contains datablock(s) I. DOI: 10.1107/S2056989024008806/yy2011Isup3.hkl

CCDC reference: 2270103

Additional supporting information:  crystallographic information; 3D view; checkCIF report

## Figures and Tables

**Table 1:** Hydrogen-bond geometry (Å, °)

*D*—H⋯*A*	*D*—H	H⋯*A*	*D*⋯*A*	*D*—H⋯*A*
C12—H12*B*⋯O1^i^	0.98	2.44	3.339 (7)	152
C12—H12*C*⋯O3^ii^	0.98	2.36	3.333 (7)	169
C12—H12*A*⋯O2^iii^	0.98	2.62	3.596 (8)	174

Symmetry codes: (i) 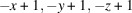; (ii) 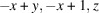; (iii) 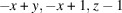.

**Table 2:** Experimental details

Crystal data	
Chemical formula	[Zn(C_4_H_6_N_2_)_6_](NO_3_)_2_
*M*_r_	682.04
Crystal system, space group	Trigonal, *P* 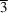
Temperature (K)	173
*a*, *c* (Å)	19.1227 (10), 7.4770 (5)
*V* (Å^3^)	2367.9 (3)
*Z*	3
Radiation type	Mo *K*α
μ (mm^−1^)	0.84
Crystal size (mm)	0.45 × 0.34 × 0.23

Data collection	
Diffractometer	Bruker D8 Venture Photon CCD area detector
Absorption correction	Multi-scan (*SADABS*; Krause *et al.*, 2015 ▸)
*T*_min_, *T*_max_	0.660, 0.745
No. of measured, independent and observed [*I* > 2σ(*I*)] reflections	119969, 3247, 3073
*R*_int_	0.040
(sin θ/λ)_max_ (Å^−1^)	0.626

Refinement	
*R*[*F*^2^ > 2σ(*F*^2^)], *wR*(*F*^2^), *S*	0.032, 0.090, 1.12
No. of reflections	3247
No. of parameters	208
H-atom treatment	H-atom parameters constrained
Δρ_max_, Δρ_min_ (e Å^−3^)	0.51, −0.36

Computer programs: *APEX3*, *SAINT-Plus* and *XPREP* (Bruker 2016 ▸), *SHELXT2014* (Sheldrick, 2015*a* ▸), *SHELXL2018/3* (Sheldrick, 2015*b* ▸), *ORTEP-3 for Windows* and *WinGX* publication routines (Farrugia, 2012 ▸) and *PLATON* (Spek, 2020 ▸).

## supplementary crystallographic information

### Hexakis(1-methyl-1H-imidazole-κN3)zinc(II) dinitrate Crystal data

**Table:**

[Zn(C_4_H_6_N_2_)_6_](NO_3_)_2_	*D*_x_ = 1.435 Mg m^−^^3^
*M**_r_* = 682.04	Mo *K*α radiation, λ = 0.71073 Å
Trigonal, *P*3	Cell parameters from 9720 reflections
*a* = 19.1227 (10) Å	θ = 3.3–26.2°
*c* = 7.4770 (5) Å	µ = 0.84 mm^−^^1^
*V* = 2367.9 (3) Å^3^	*T* = 173 K
*Z* = 3	Block, colourless
*F*(000) = 1068	0.45 × 0.34 × 0.23 mm

### Hexakis(1-methyl-1H-imidazole-κN3)zinc(II) dinitrate Data collection

**Table:**

Bruker D8 Venture Photon CCD area detector diffractometer	3073 reflections with *I* > 2σ(*I*)
Graphite monochromator	*R*_int_ = 0.040
ω scans	θ_max_ = 26.4°, θ_min_ = 2.7°
Absorption correction: multi-scan (SADABS; Krause *et al.*, 2015)	*h* = −23→23
*T*_min_ = 0.660, *T*_max_ = 0.745	*k* = −23→23
119969 measured reflections	*l* = −9→9
3247 independent reflections	

### Hexakis(1-methyl-1H-imidazole-κN3)zinc(II) dinitrate Refinement

**Table:**

Refinement on *F*^2^	Primary atom site location: dual - dual-space method e.g. SHELXD
Least-squares matrix: full	Secondary atom site location: dual - dual-space method e.g. SHELXD
*R*[*F*^2^ > 2σ(*F*^2^)] = 0.032	Hydrogen site location: mixed
*wR*(*F*^2^) = 0.090	H-atom parameters constrained
*S* = 1.12	*w* = 1/[σ^2^(*F*_o_^2^) + (0.0449*P*)^2^ + 1.2961*P*] where *P* = (*F*_o_^2^ + 2*F*_c_^2^)/3
3247 reflections	(Δ/σ)_max_ < 0.001
208 parameters	Δρ_max_ = 0.51 e Å^−^^3^
0 restraints	Δρ_min_ = −0.36 e Å^−^^3^
0 constraints	

### Hexakis(1-methyl-1H-imidazole-κN3)zinc(II) dinitrate Special details

**Table:**

Experimental. Absorption corrections were made using the program SADABS (Sheldrick, 1996) Intensity data were determined on a Bruker Venture D8 Photon CMOS diffractometer with graphite-monochromated Mo Ka_1_ (λ = 0.71073 Å) radiation at 173 K using an Oxford Cryostream 600 cooler. Data reduction was carried out using the program SAINT+, version 6.02 (Bruker, 2016) and empirical absorption corrections were made using SADABS (Bruker 2016) Space group assignments was made using *XPREP* (Bruker, 2016). The structure was solved in the *WinGX* (Farrugia, 2012) Suite of programs, using intrinsic phasing through *SHELXT* (Sheldrick, 2015a) and refined using full-matrix least-squares/difference Fourier techniques on F^2^ using *SHELXL2017* (Sheldrick, 2015b). Diagrams and publication material were generated using *ORTEP-3* (Farrugia, 2012) and *PLATON* (Spek, 2020).
Geometry. All esds (except the esd in the dihedral angle between two l.s. planes) are estimated using the full covariance matrix. The cell esds are taken into account individually in the estimation of esds in distances, angles and torsion angles; correlations between esds in cell parameters are only used when they are defined by crystal symmetry. An approximate (isotropic) treatment of cell esds is used for estimating esds involving l.s. planes.
Refinement. Refined as a 2-component twin.

### Hexakis(1-methyl-1H-imidazole-κN3)zinc(II) dinitrate Fractional atomic coordinates and isotropic or equivalent isotropic displacement parameters (Å^2^)

**Table:**

	*x*	*y*	*z*	*U*_iso_*/*U*_eq_	
C1	0.35319 (17)	0.56158 (16)	0.8432 (4)	0.0370 (5)	
H1	0.397348	0.608195	0.894545	0.044*	
C2	0.32525 (18)	0.48483 (17)	0.8998 (4)	0.0408 (6)	
H2	0.345852	0.467857	0.995698	0.049*	
C3	0.25403 (19)	0.48549 (16)	0.6749 (4)	0.0434 (6)	
H3	0.214048	0.466944	0.583882	0.052*	
C4	0.2155 (3)	0.3487 (2)	0.7945 (6)	0.0742 (12)	
H4A	0.221478	0.328911	0.911228	0.111*	
H4B	0.235574	0.327455	0.700603	0.111*	
H4C	0.158422	0.330563	0.773011	0.111*	
C5	0.31191 (18)	0.77017 (19)	0.2309 (4)	0.0408 (6)	
H5	0.26636	0.723578	0.183396	0.049*	
C6	0.34153 (19)	0.84611 (19)	0.1679 (4)	0.0451 (7)	
H6	0.321104	0.862707	0.071015	0.054*	
C7	0.41433 (19)	0.84552 (17)	0.3924 (4)	0.0479 (7)	
H7	0.455942	0.864052	0.479686	0.057*	
C8	0.4553 (3)	0.9812 (2)	0.2607 (6)	0.0860 (15)	
H8A	0.510363	0.998228	0.300205	0.129*	
H8B	0.431985	1.005728	0.337839	0.129*	
H8C	0.456463	0.998554	0.136826	0.129*	
N1	0.30848 (13)	0.56202 (13)	0.7012 (3)	0.0321 (4)	
N2	0.26215 (16)	0.43720 (15)	0.7926 (3)	0.0437 (6)	
N3	0.35725 (12)	0.77013 (12)	0.3735 (3)	0.0330 (4)	
N4	0.40615 (19)	0.89350 (16)	0.2712 (4)	0.0510 (7)	
Zn1	0.333333	0.666667	0.53840 (6)	0.02973 (13)	
C9	0.11565 (17)	0.12401 (17)	0.2960 (4)	0.0397 (6)	
H9	0.129642	0.086107	0.340727	0.048*	
C10	0.14947 (19)	0.20166 (17)	0.3498 (4)	0.0472 (7)	
H10	0.192987	0.227884	0.431476	0.057*	
C11	0.06016 (17)	0.17677 (16)	0.1425 (4)	0.0394 (6)	
H11	0.027212	0.18447	0.058845	0.047*	
C12	0.1277 (3)	0.31748 (19)	0.2617 (6)	0.0669 (10)	
H12A	0.101449	0.327601	0.160283	0.1*	
H12B	0.185931	0.3554	0.257408	0.1*	
H12C	0.105592	0.324868	0.373768	0.1*	
N5	0.05952 (13)	0.10858 (12)	0.1642 (3)	0.0353 (5)	
N6	0.11268 (16)	0.23427 (14)	0.2519 (4)	0.0453 (6)	
Zn2	0	0	0	0.03046 (17)	
N7	0.65532 (18)	0.6614 (2)	0.7378 (5)	0.0581 (7)	
O1	0.6741 (2)	0.6106 (2)	0.6944 (6)	0.1096 (13)	
O2	0.6618 (3)	0.6807 (3)	0.8925 (5)	0.1172 (14)	
O3	0.6354 (3)	0.6903 (2)	0.6181 (6)	0.1214 (15)	

### Hexakis(1-methyl-1H-imidazole-κN3)zinc(II) dinitrate Atomic displacement parameters (Å^2^)

**Table:**

	*U*^11^	*U*^22^	*U*^33^	*U*^12^	*U*^13^	*U*^23^
C1	0.0432 (14)	0.0399 (13)	0.0321 (13)	0.0239 (11)	0.0013 (11)	0.0015 (11)
C2	0.0552 (16)	0.0522 (15)	0.0319 (12)	0.0395 (14)	0.0082 (12)	0.0065 (11)
C3	0.0529 (16)	0.0332 (13)	0.0378 (14)	0.0167 (12)	−0.0037 (12)	0.0044 (11)
C4	0.099 (3)	0.0314 (16)	0.080 (3)	0.0237 (18)	−0.013 (2)	0.0114 (16)
C5	0.0464 (15)	0.0511 (16)	0.0335 (13)	0.0309 (14)	−0.0040 (12)	0.0000 (13)
C6	0.0587 (17)	0.0621 (18)	0.0342 (13)	0.0449 (15)	0.0066 (13)	0.0111 (12)
C7	0.0596 (18)	0.0318 (13)	0.0474 (16)	0.0192 (13)	−0.0115 (13)	0.0062 (11)
C8	0.126 (4)	0.043 (2)	0.082 (3)	0.037 (2)	0.003 (3)	0.0250 (19)
N1	0.0334 (10)	0.0332 (11)	0.0304 (10)	0.0171 (9)	0.0050 (8)	0.0026 (8)
N2	0.0554 (15)	0.0339 (12)	0.0426 (13)	0.0230 (11)	0.0064 (11)	0.0087 (10)
N3	0.0356 (10)	0.0352 (11)	0.0307 (10)	0.0197 (9)	0.0013 (8)	0.0031 (8)
N4	0.0727 (19)	0.0420 (14)	0.0467 (14)	0.0351 (14)	0.0049 (13)	0.0130 (11)
Zn1	0.02969 (16)	0.02969 (16)	0.0298 (2)	0.01484 (8)	0	0
C9	0.0411 (14)	0.0366 (13)	0.0371 (15)	0.0160 (12)	−0.0034 (11)	−0.0042 (11)
C10	0.0530 (16)	0.0423 (15)	0.0385 (14)	0.0179 (13)	−0.0033 (13)	−0.0024 (12)
C11	0.0407 (13)	0.0328 (12)	0.0433 (14)	0.0173 (11)	0.0000 (12)	−0.0068 (11)
C12	0.083 (3)	0.0314 (16)	0.081 (3)	0.0242 (17)	−0.006 (2)	−0.0153 (16)
N5	0.0340 (11)	0.0311 (10)	0.0395 (12)	0.0153 (9)	0.0015 (9)	−0.0015 (9)
N6	0.0497 (14)	0.0293 (12)	0.0493 (14)	0.0141 (11)	0.0028 (11)	−0.0058 (10)
Zn2	0.0271 (2)	0.0271 (2)	0.0372 (4)	0.01354 (10)	0	0
N7	0.0479 (16)	0.0584 (17)	0.0692 (19)	0.0275 (14)	0.0088 (14)	0.0225 (14)
O1	0.108 (3)	0.088 (2)	0.147 (4)	0.059 (2)	−0.004 (3)	−0.018 (2)
O2	0.156 (4)	0.150 (4)	0.0653 (18)	0.091 (3)	0.029 (2)	0.016 (2)
O3	0.131 (3)	0.113 (3)	0.133 (3)	0.071 (3)	−0.051 (3)	0.016 (3)

### Hexakis(1-methyl-1H-imidazole-κN3)zinc(II) dinitrate Geometric parameters (Å, º)

**Table:**

C1—C2	1.355 (4)	C8—H8A	0.98
C1—N1	1.365 (3)	C8—H8B	0.98
C1—H1	0.95	C8—H8C	0.98
C2—N2	1.352 (4)	N1—Zn1	2.182 (2)
C2—H2	0.95	N3—Zn1	2.177 (2)
C3—N1	1.319 (3)	C9—C10	1.351 (4)
C3—N2	1.339 (4)	C9—N5	1.376 (4)
C3—H3	0.95	C9—H9	0.95
C4—N2	1.467 (4)	C10—N6	1.363 (4)
C4—H4A	0.98	C10—H10	0.9486
C4—H4B	0.98	C11—N5	1.308 (3)
C4—H4C	0.98	C11—N6	1.335 (4)
C5—C6	1.352 (4)	C11—H11	0.95
C5—N3	1.375 (3)	C12—N6	1.471 (4)
C5—H5	0.95	C12—H12A	0.98
C6—N4	1.351 (4)	C12—H12B	0.98
C6—H6	0.95	C12—H12C	0.98
C7—N3	1.310 (3)	N5—Zn2	2.179 (2)
C7—N4	1.353 (4)	N7—O2	1.202 (5)
C7—H7	0.95	N7—O3	1.209 (4)
C8—N4	1.458 (4)	N7—O1	1.234 (5)
			
C2—C1—N1	109.9 (2)	N3—Zn1—N1^i^	88.74 (8)
C2—C1—H1	125	N3^i^—Zn1—N1^i^	179.34 (8)
N1—C1—H1	125	N3^ii^—Zn1—N1^i^	88.28 (8)
N2—C2—C1	106.3 (2)	N1^ii^—Zn1—N1^i^	91.89 (8)
N2—C2—H2	126.9	N3—Zn1—N1	179.34 (9)
C1—C2—H2	126.9	N3^i^—Zn1—N1	88.28 (8)
N1—C3—N2	111.6 (3)	N3^ii^—Zn1—N1	88.73 (8)
N1—C3—H3	124.2	N1^ii^—Zn1—N1	91.89 (8)
N2—C3—H3	124.2	N1^i^—Zn1—N1	91.89 (8)
N2—C4—H4A	109.5	C10—C9—N5	110.0 (3)
N2—C4—H4B	109.5	C10—C9—H9	124.8
H4A—C4—H4B	109.5	N5—C9—H9	125.1
N2—C4—H4C	109.5	C9—C10—N6	105.7 (3)
H4A—C4—H4C	109.5	C9—C10—H10	125.7
H4B—C4—H4C	109.5	N6—C10—H10	128.4
C6—C5—N3	110.2 (3)	N5—C11—N6	111.9 (3)
C6—C5—H5	124.9	N5—C11—H11	124
N3—C5—H5	124.9	N6—C11—H11	124
N4—C6—C5	105.8 (2)	N6—C12—H12A	109.5
N4—C6—H6	127.1	N6—C12—H12B	109.5
C5—C6—H6	127.1	H12A—C12—H12B	109.5
N3—C7—N4	111.0 (3)	N6—C12—H12C	109.5
N3—C7—H7	124.5	H12A—C12—H12C	109.5
N4—C7—H7	124.5	H12B—C12—H12C	109.5
N4—C8—H8A	109.5	C11—N5—C9	104.9 (2)
N4—C8—H8B	109.5	C11—N5—Zn2	128.3 (2)
H8A—C8—H8B	109.5	C9—N5—Zn2	125.97 (18)
N4—C8—H8C	109.5	C11—N6—C10	107.5 (2)
H8A—C8—H8C	109.5	C11—N6—C12	125.5 (3)
H8B—C8—H8C	109.5	C10—N6—C12	127.1 (3)
C3—N1—C1	105.0 (2)	N5^iii^—Zn2—N5^iv^	180.00 (9)
C3—N1—Zn1	128.56 (18)	N5^iii^—Zn2—N5	88.61 (8)
C1—N1—Zn1	126.11 (18)	N5^iv^—Zn2—N5	91.39 (8)
C3—N2—C2	107.3 (2)	N5^iii^—Zn2—N5^v^	91.39 (8)
C3—N2—C4	126.1 (3)	N5^iv^—Zn2—N5^v^	88.61 (8)
C2—N2—C4	126.5 (3)	N5—Zn2—N5^v^	180
C7—N3—C5	105.2 (2)	N5^iii^—Zn2—N5^vi^	88.61 (8)
C7—N3—Zn1	128.32 (18)	N5^iv^—Zn2—N5^vi^	91.39 (8)
C5—N3—Zn1	126.43 (19)	N5—Zn2—N5^vi^	91.39 (8)
C6—N4—C7	107.8 (3)	N5^v^—Zn2—N5^vi^	88.61 (8)
C6—N4—C8	126.0 (3)	N5^iii^—Zn2—N5^vii^	91.39 (8)
C7—N4—C8	126.1 (3)	N5^iv^—Zn2—N5^vii^	88.61 (8)
N3—Zn1—N3^i^	91.09 (8)	N5—Zn2—N5^vii^	88.61 (8)
N3—Zn1—N3^ii^	91.09 (8)	N5^v^—Zn2—N5^vii^	91.39 (8)
N3^i^—Zn1—N3^ii^	91.09 (8)	N5^vi^—Zn2—N5^vii^	180.00 (8)
N3—Zn1—N1^ii^	88.28 (8)	O2—N7—O3	125.4 (4)
N3^i^—Zn1—N1^ii^	88.73 (8)	O2—N7—O1	118.0 (4)
N3^ii^—Zn1—N1^ii^	179.34 (8)	O3—N7—O1	116.5 (4)
			
N1—C1—C2—N2	−0.4 (3)	C5—C6—N4—C7	0.3 (3)
N3—C5—C6—N4	0.5 (3)	C5—C6—N4—C8	−176.3 (4)
N2—C3—N1—C1	0.1 (3)	N3—C7—N4—C6	−1.1 (4)
N2—C3—N1—Zn1	173.38 (18)	N3—C7—N4—C8	175.5 (4)
C2—C1—N1—C3	0.2 (3)	N5—C9—C10—N6	1.0 (3)
C2—C1—N1—Zn1	−173.31 (17)	N6—C11—N5—C9	−0.2 (3)
N1—C3—N2—C2	−0.3 (3)	N6—C11—N5—Zn2	−170.42 (18)
N1—C3—N2—C4	−175.6 (3)	C10—C9—N5—C11	−0.6 (3)
C1—C2—N2—C3	0.4 (3)	C10—C9—N5—Zn2	170.0 (2)
C1—C2—N2—C4	175.7 (3)	N5—C11—N6—C10	0.8 (3)
N4—C7—N3—C5	1.4 (4)	N5—C11—N6—C12	−179.7 (3)
N4—C7—N3—Zn1	−175.52 (19)	C9—C10—N6—C11	−1.1 (3)
C6—C5—N3—C7	−1.2 (3)	C9—C10—N6—C12	179.4 (3)
C6—C5—N3—Zn1	175.81 (18)		

Symmetry codes: (i) −*y*+1, *x*−*y*+1, *z*; (ii) −*x*+*y*, −*x*+1, *z*; (iii) *x*−*y*, *x*, −*z*; (iv) −*x*+*y*, −*x*, *z*; (v) −*x*, −*y*, −*z*; (vi) −*y*, *x*−*y*, *z*; (vii) *y*, −*x*+*y*, −*z*.

### Hexakis(1-methyl-1H-imidazole-κN3)zinc(II) dinitrate Hydrogen-bond geometry (Å, º)

**Table:**

*D*—H···*A*	*D*—H	H···*A*	*D*···*A*	*D*—H···*A*
C12—H12*B*···O1^viii^	0.98	2.44	3.339 (7)	152
C12—H12*C*···O3^ii^	0.98	2.36	3.333 (7)	169
C12—H12*A*···O2^ix^	0.98	2.62	3.596 (8)	174

Symmetry codes: (ii) −*x*+*y*, −*x*+1, *z*; (viii) −*x*+1, −*y*+1, −*z*+1; (ix) −*x*+*y*, −*x*+1, *z*−1.
